# Supplementary material for: Comparative site-specific N-glycoproteome analysis reveals aberrant N-glycosylation and gives insights into mannose-6-phosphate pathway in cancer
Source: Commun Biol. 2023 Jan 13;6:48. doi: 10.1038/s42003-023-04439-4 (PMC9839730; doi:10.1038/s42003-023-04439-4)
Supplement: Supplementary file 2 — Supplementary Figures [file 42003_2023_4439_MOESM2_ESM.pdf]

Comparative site-specific N-glycoproteome analysis reveals aberrant N-glycosylation and gives insights into mannose-6-phosphate pathway in cancer

Chen et al.

Supplementary Figure 1: Uncropped and unedited blots correspond to Figure 2b

Supplementary Figure 2: Uncropped and unedited blots correspond to Figure 6c

Supplementary Figure 3: Uncropped and unedited blots correspond to Figure 7c

Supplementary Figure 4: Uncropped and unedited blots correspond to Figure 7g

Supplementary Figure 1

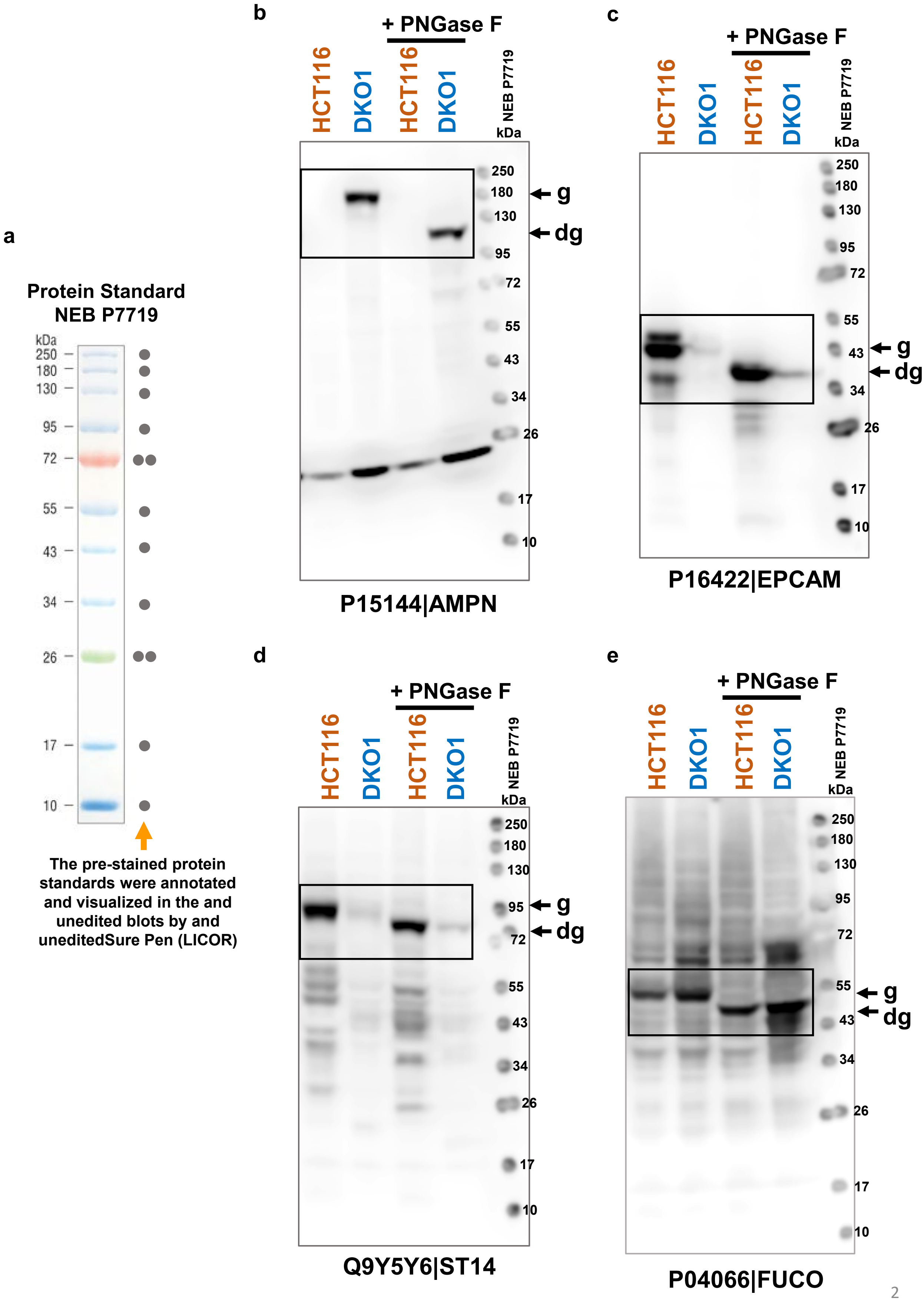

Supplementary Figure 1

f

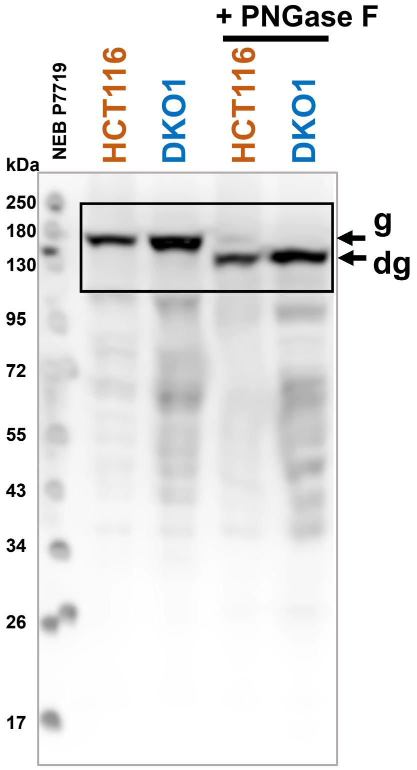

Q9Y4L1|HYOU1

g

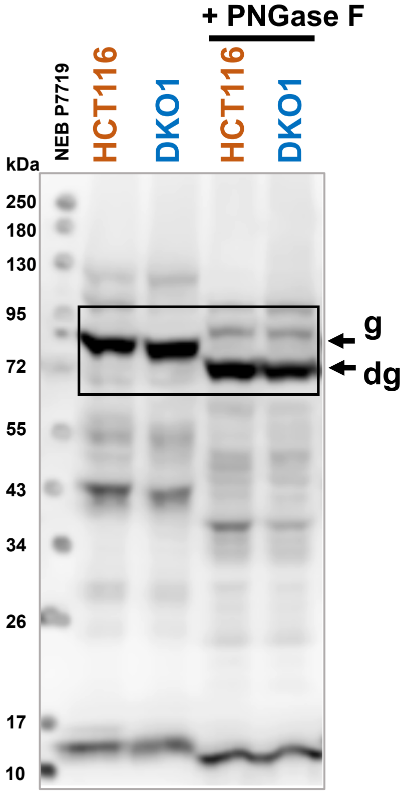

P10253|LYAG

h

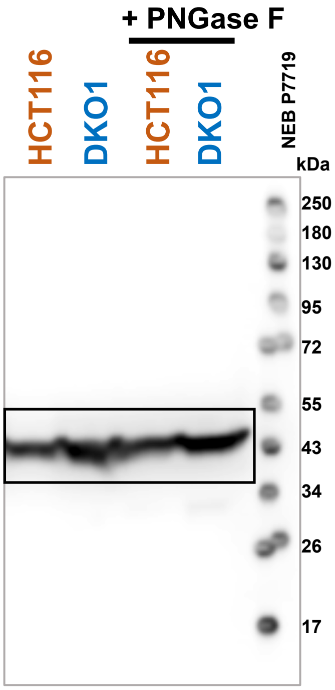

β-actin

Supplementary Figure 2

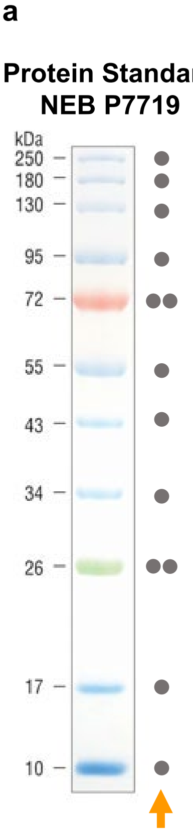

The pre-stained protein standards were annotated and visualized in the and unedited blots by and uneditedSure Pen (LICOR)

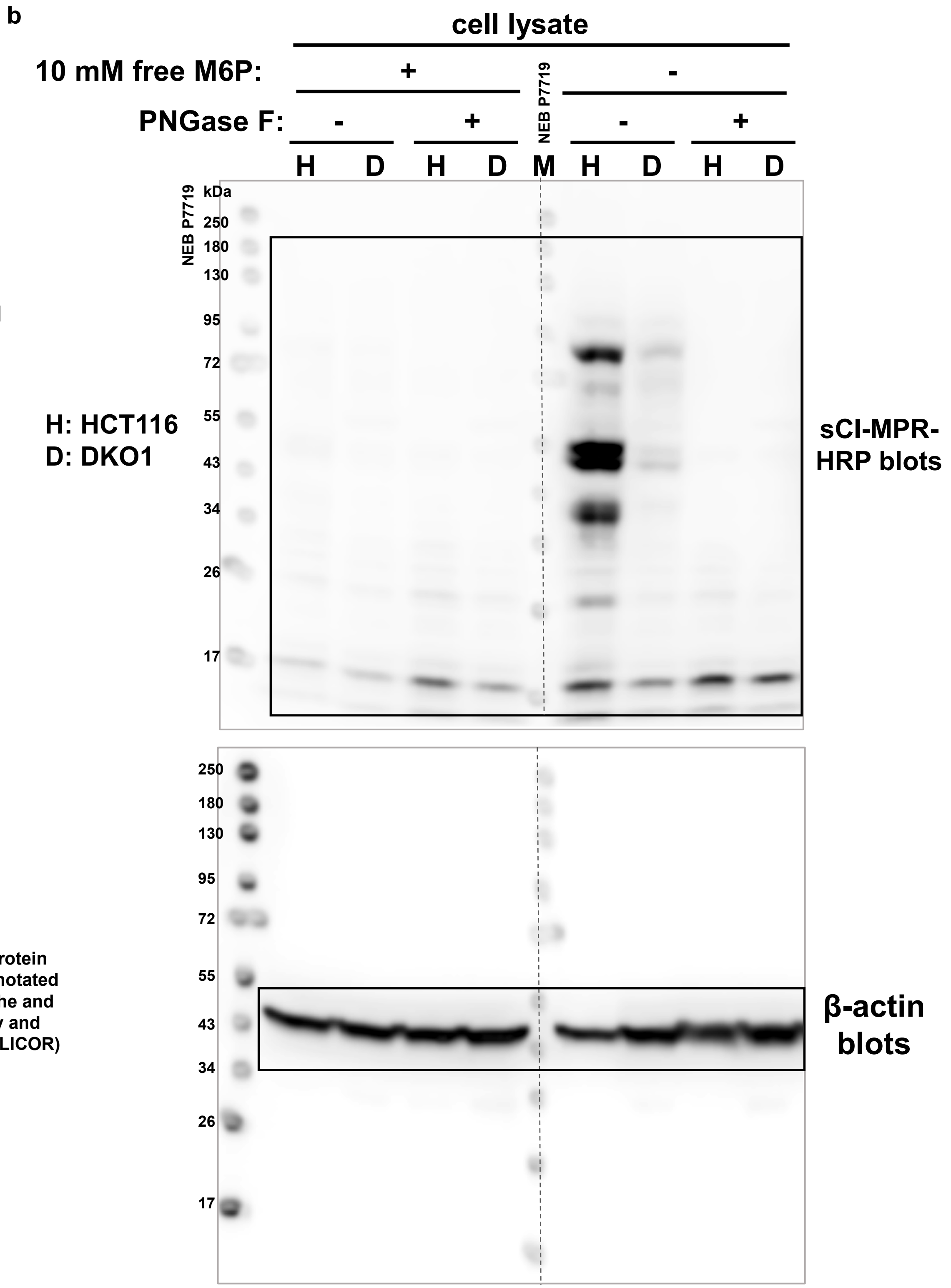

Supplementary Figure 3

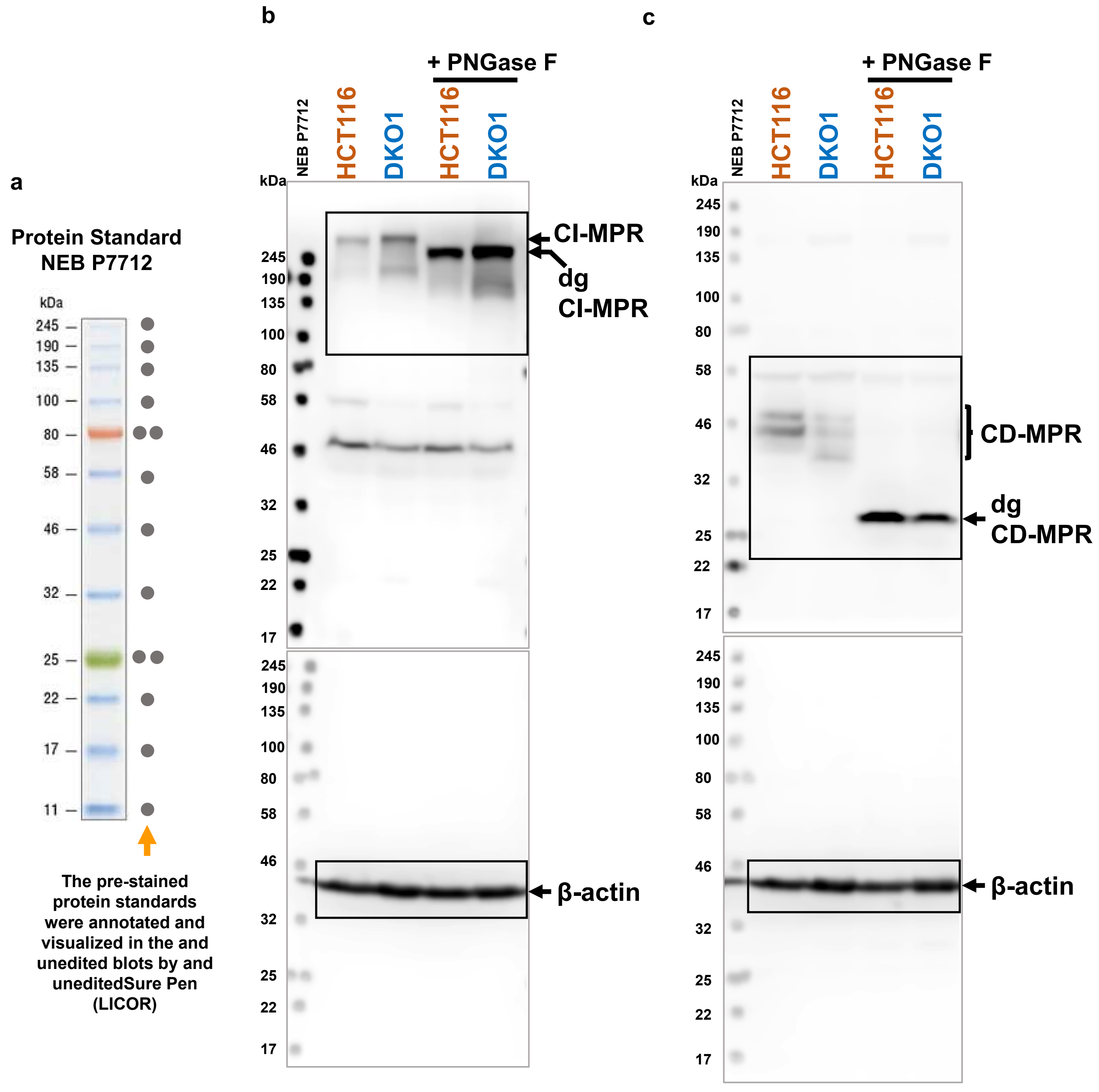

dg: deglycosylated

Supplementary Figure 4

a

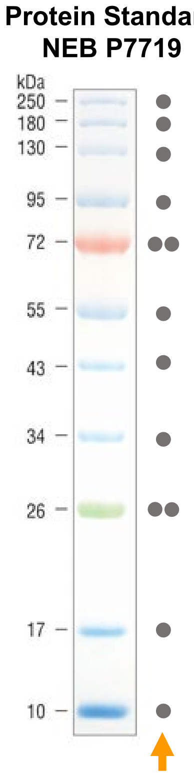

The pre-stained protein standards were annotated and visualized in the and unedited blots by and uneditedSure Pen (LICOR)

b

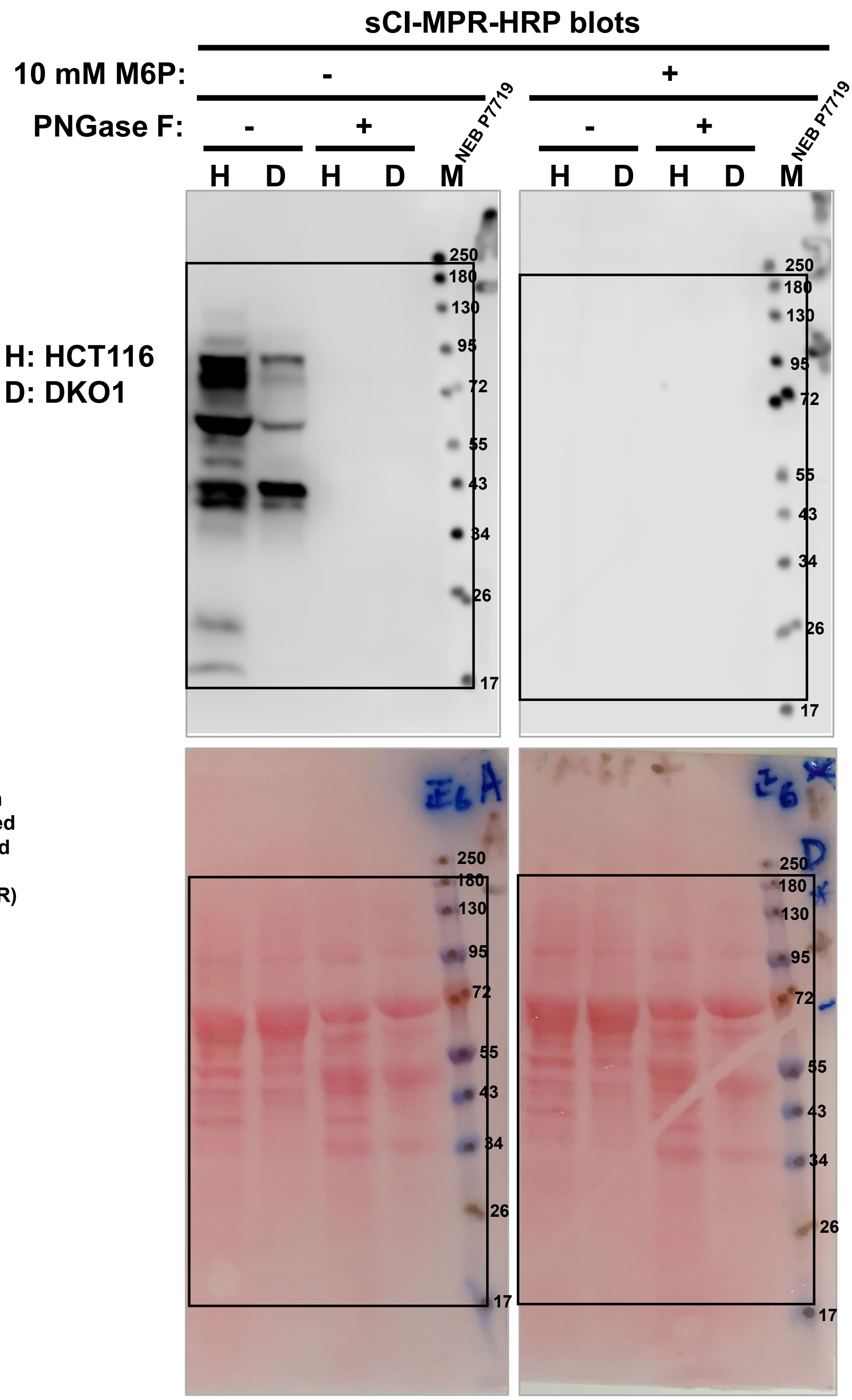

Secretion / Conditioned Medium
