# Supplementary material for: Comparative site-specific N-glycoproteome analysis reveals aberrant N-glycosylation and gives insights into mannose-6-phosphate pathway in cancer
Source: Commun Biol. 2023 Jan 13;6:48. doi: 10.1038/s42003-023-04439-4 (PMC9839730; doi:10.1038/s42003-023-04439-4)
Supplement: Supplementary file 3 — Description of Additional Supplementary Files [file 42003_2023_4439_MOESM3_ESM.docx]

# Supplemental data legend (Chen *et. al*)

**Supplemental Data 1 (two tabs)**

- **Red tab, “pre-enrichment all”**: Raw Byonic search data including N-glycopeptides and non-N- glycopeptides from pre-enrichment samples with 45 min LC. Peptides from HCT116 are highlighted in yellow and peptides from DKO1 are highlighted in blue.
- **Yellow tab, “pre-enrichment N-glyco”**: N-glycopeptides only, from the raw Byonic search data of pre-enrichment samples with 45 min LC shown in the red tab. N-glycopeptides from HCT116 are highlighted in yellow and N-glycopeptides form DKO1 are highlighted in blue.

# Supplemental Data 2 (two tabs)

- **Green tab, “Enriched 45 min LC all”**: Raw Byonic search data including N-glycopeptides and non-N-glycopeptides from Fbs1-GYR enriched samples with 45 min LC. Peptides from HCT116 are highlighted in yellow and peptides from DKO1 are highlighted in blue.
- **Orange tab, “Enriched 45 min N-glyco”**: N-glycopeptides only, from Fbs1-GYR enriched samples with 45 min LC shown in the green tab. N-glycopeptides from HCT116 are highlighted in yellow and N-glycopeptides from DKO1 are highlighted in blue.

# Supplemental Data 3 (two tabs)

- **Red tab, “All MS data score >=0”**: Byonic search data (with Byonic score >=0) including N- glycopeptides and non-N-glycopeptides from all Fbs1-GYR enriched samples (include all the MS runs with 45- and 90-min LC).
- **Orange tab, “All N-glyco score >=0”**: N-glycopeptides only, from the Byonic search data (with Byonic score >=0) from all Fbs1-GYR enriched samples (include all MS runs with 45- and 90- min LC) shown in the red tab.

# Supplemental Data 4 (three tabs)

- **Green tab, “All MS data score >=300”**: Byonic search data (with Byonic score >=300) including N-glycopeptides and non-N-glycopeptides from all Fbs1-GYR enriched samples (include all the MS runs with 45- and 90-min LC).
- **Blue tab, “All N-glyco score >=300”**: N-glycopeptides only, from the Byonic search data (with Byonic score >=300) from all Fbs1-GYR enriched samples (include all MS runs with 45- and 90- min LC) shown in the green tab.
- **Purple tab, “for Fig. 1c”**: Detailed data for Figure 1c.

# Supplemental Data 5 (four tabs)

- **Red tab, “Full summary N-glycoproteomes”**: A Pivot table to show all the site-specific N- glycosylation with detailed N-glycan compositions. The original data for this Pivot table is the Byonic search data (with Byonic score >=300) from all Fbs1-GYR enriched samples (include all MS runs with 45- and 90- min LC) shown in Blue tab of Supplemental Data 4. The green row indicates N-glycoprotein identity (Uniport # and protein name). N-glycosites in the protein are listed below N-glycoprotein identity in yellow rows. Under the N-glycosite, N-glycan compositions are listed. N-glyco PSM from DKO1 or HCT116 are listed next to protein

identities, N-glycosites, and N-glycans. Relative glycosylation abundance (RGA) of a site- specific N-glycosylation is calculated by N-glyco PSM of a site-specific N-glycosylation divided by the total N-glyco PSM of the corresponding protein.

- **Purple tab, “classified N-glycans”**: N-glycan compositions are categorized into eight N-glycan classes.
- **Yellow tab, “glycoproteomes w glycan class”**: The N-glycan class information were added to the Byonic search data (with Byonic score >=300) from all Fbs1-GYR enriched samples (include all MS runs with 45- and 90- min LC) shown in Blue tab of Supplemental Data 4.
- **Blue tab, “Simplified N-glycoproteomes”**: A Pivot table to show all the site-specific N- glycosylation with N-glycan classes. The original data for this Pivot table is the data shown in the yellow tab of Supplemental Data 5. The green row indicates N-glycoprotein identity (Uniport # and protein name). N-glycosites in the protein are listed below N-glycoprotein identity in yellow rows. Under the N-glycosite, the attached N-glycan class are listed. N-glyco PSM from DKO1 or HCT116 are listed next to protein identities, N-glycosites, and N-glycan class. Relative glycosylation abundance (RGA) of a site-specific N-glycosylation is calculated by N-glyco PSM of a site-specific N-glycosylation divided by the total N-glyco PSM of the corresponding protein.

# Supplemental Data 6 (five tabs)

- **Brown tab, “For Fig. 3a”**: The identities of the dots shown in Figure 3a.
- **Green tab, “For Fig. 3b”**: A searchable table for Figure 3b.
- **Red tab, “For Fig. 3c”**: Detailed calculation for Figure 3c.
- **Orange tab, “For Fig. 3d”**: A searchable table for Figure 3d.
- **Blue tab, “For Fig. 3e”**: A searchable table for Figure 3e.

# Supplemental Data 7 (two tabs)

- **Red tab, “For Fig. 4a”**: Detailed information for Figure 4a.
- **Yellow tab, “For Fig. 4b”**: A searchable table for Figure 4b. Relative glycosylation abundance (RGA) is calculated by specific N-glyco PSM divided by total N-glyco PSM of the corresponding protein. RGA fold change is calculated by the ratio of RGA in DKO1 to RGA in HCT116 (in blue) or the ratio of RGA in HCT116 to RGA in DKO1 (in orange). Selection criteria are listed for different N-glycan classes.

# Supplemental Data 8 (three tabs)

- **Green tab, “For Fig. 5a”**: A searchable table for Figure 5a. Relative glycosylation abundance (RGA) is calculated by PauciM N-glyco PSM divided by total N-glyco PSM of the corresponding protein. The paucimannose-rich N-glycoproteins that are annotated to extracellular exosome and lysosome are in red and highlighted in green, respectively.
- **Blue tab, “For Fig. 5b”**: Gene Ontology (GO) annotations of the PauciM-rich proteins using the terms of cellular components (A), biological process (B), or molecular function (C).
- **Red tab, “For Fig. 5c”**: A searchable table for Figure 5c. Relative glycosylation abundance (RGA) is calculated by PauciM N-glyco PSM divided by total N-glyco PSM of the corresponding protein. RGA fold change is calculated by the ratio of RGA in DKO1 to RGA in HCT116.

Q9Y4L1|HYOU1 with a very large number of the total N-glyco PSM (2659 in DKO1 and 1269 in HCT116) and a 169.4-fold change in paucimannose glycosylation, is highlighted in purple.

# Supplemental Data 9 (one tab)

- **Yellow tab, “For Fig. 6a”**: A searchable table for Figure 6a. Relative glycosylation abundance (RGA) is calculated by M6P N-glyco PSM divided by total N-glyco PSM of the corresponding protein.

# Supplemental Data 10 (one tab)

# The source data of Figure 1c, 2c, 4a, 6a, 6d, 7d, 7e, and 7h.
