# Supplementary material for: Comparative site-specific N-glycoproteome analysis reveals aberrant N-glycosylation and gives insights into mannose-6-phosphate pathway in cancer
Source: Commun Biol. 2023 Jan 13;6:48. doi: 10.1038/s42003-023-04439-4 (PMC9839730; doi:10.1038/s42003-023-04439-4)
Supplement: Supplementary file 14 — nr-reporting-summary [file 42003_2023_4439_MOESM14_ESM.pdf]

Corresponding author(s): Minyong Chen
Last updated by author(s): Jan 4, 2023

Reporting Summary

Nature Portfolio wishes to improve the reproducibility of the work that we publish. This form provides structure for consistency and transparency in reporting. For further information on Nature Portfolio policies, see our Editorial Policies and the Editorial Policy Checklist.

Statistics

For all statistical analyses, confirm that the following items are present in the figure legend, table legend, main text, or Methods section.

n/a	Confirmed	
☐	☒	The exact sample size (*n*) for each experimental group/condition, given as a discrete number and unit of measurement
☐	☒	A statement on whether measurements were taken from distinct samples or whether the same sample was measured repeatedly
☐	☒	The statistical test(s) used AND whether they are one- or two-sided<br>*Only common tests should be described solely by name; describe more complex techniques in the Methods section.*
☒	☐	A description of all covariates tested
☒	☐	A description of any assumptions or corrections, such as tests of normality and adjustment for multiple comparisons
☒	☐	A full description of the statistical parameters including central tendency (e.g. means) or other basic estimates (e.g. regression coefficient) AND variation (e.g. standard deviation) or associated estimates of uncertainty (e.g. confidence intervals)
☒	☐	For null hypothesis testing, the test statistic (e.g. *F*, *t*, *r*) with confidence intervals, effect sizes, degrees of freedom and *P* value noted<br>*Give P values as exact values whenever suitable.*
☒	☐	For Bayesian analysis, information on the choice of priors and Markov chain Monte Carlo settings
☒	☐	For hierarchical and complex designs, identification of the appropriate level for tests and full reporting of outcomes
☒	☐	Estimates of effect sizes (e.g. Cohen's *d*, Pearson's *r*), indicating how they were calculated

Our web collection on statistics for biologists contains articles on many of the points above.

Software and code

Policy information about availability of computer code

Data collection	Byonic, a MS/MS search engine, which is embedded as a node in Byos from Protein Metrics, was used for N-glycopeptide identification.
Data analysis	Microsoft Excel and GraphPad Prism were used for data analysis

For manuscripts utilizing custom algorithms or software that are central to the research but not yet described in published literature, software must be made available to editors and reviewers. We strongly encourage code deposition in a community repository (e.g. GitHub). See the Nature Portfolio guidelines for submitting code & software for further information.

Data

Policy information about availability of data

All manuscripts must include a data availability statement. This statement should provide the following information, where applicable:

- Accession codes, unique identifiers, or web links for publicly available datasets
- A description of any restrictions on data availability
- For clinical datasets or third party data, please ensure that the statement adheres to our policy

The uncropped and unedited blot images are available in Supplementary Figures 1-4. The source data of Figure 1c, 2c, 4a, 6a, 6d, 7d, 7e, and 7h are provided in Supplemental Data 7. The mass spectrometry proteomics data have been deposited to the ProteomeXchange Consortium via the PRIDE partner repository (http://www.ebi.ac.uk/pride) with the dataset identifier PXD035301.

Human research participants

Policy information about studies involving human research participants and Sex and Gender in Research.

Reporting on sex and gender	N/A
Population characteristics	N/A
Recruitment	N/A
Ethics oversight	N/A

Note that full information on the approval of the study protocol must also be provided in the manuscript.

Field-specific reporting

Please select the one below that is the best fit for your research. If you are not sure, read the appropriate sections before making your selection.

☒ Life sciences ☐ Behavioural & social sciences ☐ Ecological, evolutionary & environmental sciences

For a reference copy of the document with all sections, see nature.com/documents/nr-reporting-summary-flat.pdf

Life sciences study design

All studies must disclose on these points even when the disclosure is negative.

Sample size	In this study, three biological replicates (cells with three different passages) for each cell line (HCT116 or DKO1) were used.
Data exclusions	N/A
Replication	Three biological replicates (cells with three different passages) for each cell line (HCT116 or DKO1) were used. For N-glycoproteomic studies, two MS/MS runs were performed for each biological replicate, thus a total of six MS/MS runs for each cell line was analyzed. For Western blots to analyze the protein abundance, both relative quantifications of the glycosylated and de-glycosylated proteins were included in the statistical analysis (n=6 for each cell line). For M6P western blots, the sample size is 3 (three biological replicates for each cell line) for statistical analysis. For qRT-PCR analysis of mRNA levels of CI-MPR and CD-MPR, the statistical sample size is 24 (8 technical replicates for each biological replicate).
Randomization	N/A
Blinding	N/A

Reporting for specific materials, systems and methods

We require information from authors about some types of materials, experimental systems and methods used in many studies. Here, indicate whether each material, system or method listed is relevant to your study. If you are not sure if a list item applies to your research, read the appropriate section before selecting a response.

Materials & experimental systems

n/a	Involved in the study	
☐	☒	Antibodies
☐	☒	Eukaryotic cell lines
☒	☐	Palaeontology and archaeology
☒	☐	Animals and other organisms
☒	☐	Clinical data
☒	☐	Dual use research of concern

Methods

n/a	Involved in the study	
☒	☐	ChIP-seq
☒	☐	Flow cytometry
☒	☐	MRI-based neuroimaging

Antibodies

Antibodies used	Anti-EPCAM (AF960) and Anti-AMPN (AF3815) antibodies were from R&D Systems. Antibodies against β-actin (sc-47778), CD-MPR (sc-365196), ST14 (sc-365482), LYAG (sc-373745), HYOU1 (sc-398224), and FUCO (sc-365496) were from Santa Cruz Biotechnology (SCBT). CI-MPR antibody (D8Z3J, #15128S) were purchased from Cell Signaling Technology (CST)

Validation	Anti-EPCAM (AF960): https://www.rndsystems.com/products/human-epcam-trop-1-antibody_af960<br>Anti-AMPN (AF3815): https://www.rndsystems.com/products/human-aminopeptidase-n-cd13-antibody_af3815<br>Anti-β-actin (sc-47778), https://www.scbt.com/p/beta-actin-antibody-c4?requestFrom=search<br>Anti-CD-MPR (sc-365196), https://www.scbt.com/p/cd-mpr-antibody-h-7?requestFrom=search<br>Anti-ST14 (sc-365482), https://www.scbt.com/p/matriptase-antibody-d-7?requestFrom=search<br>Anti-LYAG (sc-373745), https://www.scbt.com/p/lyag-antibody-g-7?requestFrom=search<br>Anti-HYOU1 (sc-398224), https://www.scbt.com/p/orp150-antibody-a-3?requestFrom=search<br>Anti-FUCO (sc-365496), https://www.scbt.com/p/fuca1-antibody-g-12?requestFrom=search<br>Anti-CI-MPR antibody (D8Z3J, #15128S), https://www.cellsignal.com/products/primary-antibodies/igf-ii-receptor-ci-m6pr-d8z3j-rabbit-mab/15128<br>Soluble CI-MPR (sCI-MPR) (Recombinant human IGF-II R/IGF2R (aa 43-1365) Protein, CF, Cat #: 6418-GR), https://www.rndsystems.com/products/recombinant-human-igf-ii-r-igf2r-aa-43-1365-protein-cf_6418-gr<br><br>The N-glycoproteins we detected using the antibodies listed above were also confirmed by PNGase F deglycosylation and right molecular weights of deglycosylated forms.<br>Mannose-6-phosphate (M6P) western blots was validated by adding 10 mM free M6P in parallel western blots to compete off soluble CI-MPR binding to M6P on the cell lysate proteins blotted on the membranes.

Eukaryotic cell lines

Policy information about cell lines and Sex and Gender in Research

Cell line source(s)	HCT116 and its isogenic DNMT1 and DNMT3b double knockout cell line (DKO1) were obtained from Bert Vogelstein's lab.
Authentication	Other New England Biolabs researchers routinely perform next generation sequencing of these cell lines. These experiments serve to authenticate the cell lines studied here.
Mycoplasma contamination	N/A
Commonly misidentified lines<br>(See ICLAC register)	N/A
